# Factors Related to Mortality in Patients with Fournier’s Gangrene or Necrotising Fasciitis; a 10-year Cross-Sectional Study

**DOI:** 10.22037/aaem.v9i1.1123

**Published:** 2021-04-17

**Authors:** Anahita Ansari Djafari, Amirhossein Rahavian, Babak Javanmard, Saeed Montazeri, Vahid Shahabi, Seyyed Ali Hojjati, Saleh Ghiasy, Ramin Hamidi, Jalaluddin Khoshnevis

**Affiliations:** 1 Laser Application in Medical Sciences Research Center, Shohada-e-Tajrish Hospital, Shahid Beheshti University of Medical Sciences, Tehran, Iran.; 2 Andrology Research Center, Yazd Reproductive Sciences Institute, Shahid Sadoughi University of Medical Sciences, Yazd, Iran.; 3 Urology Department, Shohada-e-Tajrish Hospital, School of Medicine, Shahid Beheshti University of Medical Sciences, Tehran, Iran.; 4 School of Medicine, 501 Hospital (Imam Reza), AJA University of Medical Science, Tehran, Iran.; 5 Clinical Research Development Unit, Shohada-e-Tajrish Hospital, Shahid Beheshti University of Medical Sciences, Tehran, Iran.

**Keywords:** Fournier’s Gangrene, Fournier’s Gangrene Severity Index, Mortality, Thrombocytopenia

## Abstract

**Introduction:**

Fournier’s gangrene (FG) is a life-threatening disease, even with early diagnosis and administration of vigorous treatment, its mortality rate is high. This study aimed to evaluate the factors relate to mortality in patients with FG or necrotising fasciitis managed in a referral center.

**Methods:**

This retrospective cross-sectional study was conducted on patients managed in a tertiary referral center, Tehran, Iran, from March 2009 to March 2019, with diagnosis of FG or necrotising fasciitis. The correlation between different demographic and clinical parameters with mortality was analysed and reported.

**Results:**

73 cases with the mean age of 59.1 ± 15.8 (range: 25 – 88) years were studied (87.7% male). 21 (28.8%) patients died. Escherichia coli (26 cases, 35.6%) was the most frequent microorganism in cultures. Non-survived cases had higher mean age (p = 0.01), higher frequency of hyperlipidaemia (p = 0.02), immunosuppression (p < 0.001), longer hospital stay (p=0.02), lower blood pressure (p=0.01), and lower platelet count (p=<0.001). Based on multivariate analysis, age (p = 0.015; Odds: 0.88 (0.79-0.97)), haematocrit level (p = 0.01; Odds: 1.27 (1.04-1.55)), platelet count (p = 0.03; Odds: 10.11 (1.14-89.35)), and immunosuppression (p = 0.01; Odds: 0.01 (0.0-0.54)) were independent related factors of mortality.

**Conclusions:**

The rate of mortality due to FG and necrotizing fasciitis was 28.8%. Based on multivariate analysis, the independent related factors of mortality were older age, lower haematocrit level and platelet count, and presence of immunosuppression.

## Introduction

Fournier’s gangrene (FG) is a rapidly progressive, life-threatening necrotising infection of soft tissues, which typically involves the external genitalia and perineal area, but can also affect the abdominal wall and thighs (1). 

The disease mortality rate remains high despite early surgical interventions and advances in intensive care and medical treatment, due to its rapidly progressive nature (2-4). In recent decades, several studies published in the medical literature have examined the diverse scoring systems used to predict the risk of mortality in these patients (5, 6). One of these scores is Fournier’s Gangrene Severity Index Score (FGSI). FGSI incorporates nine clinical and para-clinical parameters, including temperature, heart rate, respiratory rate, serum sodium, serum potassium, serum creatinine, leukocyte count, haematocrit and arterial blood level of bicarbonate (7). Some studies confirmed the efficacy of FGSI (8) and Lin et al. introduced simplified FGSI including creatinine, hematocrit and serum potassium (9). In contrast, some studies concluded that this score cannot predict the mortality rate (10). Moreover, it seems that other parameters and laboratory data are also capable of effectively predicting the prognosis in such septic patients (11). The medical literature has also reported several other clinical factors related to the increased mortality risk in patients with FG. Some of these factors include primary anorectal cancer, immunosuppression, senectitude, delay in treatment initiation, and hepatic dysfunction (12, 13).

The aim of this study was to evaluate the factors related to mortality in patients with FG or necrotising fasciitis managed in a referral Urology Center, over the past ten years.

## Methods

***Study design and settings***

This retrospective cross-sectional study was conducted on patients managed in a tertiary referral center, Tehran, Iran, from March 2009 to March 2019, with diagnosis of Fournier’s gangrene or necrotising fasciitis. The correlation between different demographic and clinical parameters with mortality was analysed and reported. The ethical committee of the hospital approved this study and permitted us to review patients' medical data (IR.SBMU.RETECH.REC.1398.476). The patients' health records were anonymised.

***Data gathering***

The source of data was the medical profile of patients, which was searched using terms such as Fournier’s gangrene, soft tissue infection, and necrotising fasciitis. The collected medical data included sex, age, co-morbidities (diabetes mellitus, cerebrovascular accidents, malignancies, urinary incontinence, bed ridden), laboratory findings, duration of hospital stay, antibiotics used in the treatment, immune system status and the final outcome. Disease diagnosis was based on the symptoms of pain, erythema, ulcers, swelling, crepitus, necrosis, purulent discharge and later confirmation by tissue inspection in the operating room. Also, vital signs (pulse rate, respiratory rate, blood pressure and temperature) were assessed for signs of systemic inflammatory response syndrome. 

In addition, Fournier's Gangrene Severity Index (FGSI), which incorporates nine variables (temperature, heart rate, respiratory rate, serum sodium, serum potassium, serum creatinine, haematocrit, white blood cell count, and serum bicarbonate), was calculated for all patients (3). 

The patients were classified, according to the extent of FG, in to the following groups: 1) patients with gangrene confined to the scrotum, 2) patients with penile and scrotal gangrene, 3) patient with scrotal gangrene extending to the perineum, 4) patient with scrotal gangrene extending to the abdominal wall or thighs. 

Patients with solitary abscess as well as those with soft tissue infection without perineal or scrotal involvement were excluded from the study. Patients with incomplete data were also omitted.

Mortality was described as disease-related death during the hospital stay. 

Two urology residents read the records and gathered the data and finally, the corresponding author verified the data.

**Statistical analysis**

Statistical analysis was performed using Statistical Package for the Social Sciences version 26. First, the normality of data was checked and independent t-test and Mann-Whitney test were used according to the type of hypothesis. Quantitative outcomes were shown using descriptive statistics (mean ± standard deviation) and qualitative variables were reported as frequency (%). Regression analysis was performed to determine the association of risk factors and mortality of FG. A P-value less than 0.05 was considered statistically significant.

## Results

106 patients with FG or necrotising fasciitis were admitted to the studied hospital during the study period. 33 cases were excluded because of insufficient data. 73 cases with the mean age of 59.1 ± 15.8 (range: 25 – 88) years were studied (87.7% male). 21 (28.8%) patients died. 

The most commonly involved area was perineum. All cases were treated with surgical debridement of necrotic tissues, in 21(28.8%) of whom debridement was extensive enough to necessitate additional tissue reconstruction (flap (3 cases), tissue graft (6 cases), and secondary suturing (12 cases)). 

 The results of wound culture were available for 52 patients and Escherichia coli (26 cases, 35.6%) was the most frequent microorganism. All four patients whose wound cultures were positive for Klebsiella, Proteus, Pseudomonas and Acinetobacter died.

Non-survived cases had higher mean age (p = 0.01), higher frequency of hyperlipidaemia (p = 0.02), immunosuppression (p < 0.001), longer hospital stay (p=0.02), lower blood pressure (p=0.01), and lower platelet count (p=<0.001). Seven patients had platelet counts of <50000/mm³, six of whom died.

Regarding FGSI variables, non-survived patients had lower serum Haematocrit (p=0.0), lower serum bicarbonate (p=0.0), and higher respiratory rate (p=0.03).

The mean FGSI score was 8.7±3.5 in dead and 3.5 ± 2.9 in survived cases (p < 0.0001). 50 (96.2%) survived cases and 11 (42.4%) dead cases had FGSI ≤ 9. 10 (47.6%) non-survived cases had FGSI > 9. Table 1 compares the baseline characteristics of participants between survived and non-survived cases.

Table 2 shows the factors related to mortality based on the multivariate analysis. Based on this analysis, age, haematocrit level, platelet count, and immunosuppression were independent factors related to mortality.

**Table 1:** Comparing the baseline characteristics between survived and non-survived patients with Fournier’s gangrene or necrotising fasciitis

**Variables**	**Died (n = 21)**	**Survived (n = 52)**	**P value**
**Sex **			
Male	16 (76.2)	48(92.4)	0.07
Female	5 (23.8)	4(7.6)	
**Age (year)**			
Mean ± SD	66.2 ± 11.1	56.1 ± 16.5	0.01
**Medical history**			
Diabetes mellitus	11(52.3)	23(44.2)	0.35
Hyperlipidaemia	4(19.0)	1(1.9)	0.02
Immunosuppression	11(52.3)	7(13.4)	<0.001
Colorectal or urological surgeries	4(19.0)	16(30.7)	0.38
**Presenting vital sign**			
Temperature (°C)	37.4 ±0.7	37.3 ±0.8	0.90
Respiratory Rate (/minute)	19.2 ±4.8	16.8 ±1.8	0.03
Pulse Rate (beats/minute)	89.2±16.8	83.8±10.9	0.10
Systolic blood pressure	114.2±16.3	124.9±17	0.01
**Laboratory findings**			
Serum Creatinine (mg/dl)	2.8 ±1.8	1.7±2.9	0.06
Serum Sodium (mM)	134.5 ±7.3	137 ±4.8	0.10
Serum Potassium(mM)	4 ±0.6	4 ±0.4	0.70
Haematocrit (%)	27.1 ±5.1	33.5 ±5.5	< 0.001
White blood cells (10³/μL)	16.1 ±8.8	15.1 ±6.7	0.6
Serum HCO_3_ (mM)	17.2 ±6.2	23 ±6.1	< 0.001
Platelet (/mm^3^)	135400±77600	256700±136800	< 0.001
**Site of involvement**			
Scrotum	1(4.7)	15(28.8)	0.30
Penis	0(0.0)	4(7.6)	
Perineum	7(33.3)	13(25.0)	
Perianal	9(42.8)	9(17.3)	
Abdomen	4(19)	11(21.1)	
**Hospital stay (days)**			
Mean ± SD	18.0 ± 22.6	10.1 ± 7.1	0.02

Data are presented as mean ± standard deviation or frequency (%).

**Table 2:** Independent factors related to mortality in patients with Fournier’s Gangrene or Necrotising Fasciitis

**Independent factors**	**P-value**	Odds ratio (95% CI*)
Age	0.015	0.88 (0.79-0.97)
Haematocrit	0.01	1.27 (1.04-1.55)
Immunosuppression	0.03	10.11 (1.14-89.35)
Platelet > 50000	0.02	0.01 (0.0-0.54)

*CI: confidence interval.

## Discussion

This study investigated 73 cases of FG or necrotising fasciitis who were hospitalised in our center over a 10-year period. The rate of mortality amongst our FG patients was 28.8%. Based on multivariate analysis, the independent related factors of mortality were older age, lower haematocrit level and platelet count, and presence of immunosuppression.

The mean age of survivors and deceased patients was significantly different; also, in multivariate analysis, we showed that age had a direct effect on mortality of the patients. Some published studies show similar results (7), whilst the result of others is contradictory to ours (4, 14).

Tuncel et al. reported a correlation between diabetes mellitus (DM) and poor prognosis in FG patients (10), but Corcoran et al. found no similar association in their research (8). In this study, there was no significant difference between the frequency of DM amongst survivors and patients who died. The frequency of hyperlipidaemia and immunosuppression, was significantly different between survived and non-survived cases (p = 0.02 and p < 0.001, respectively).

E. coli, Streptococcus, Staphylococcus, Enterococcus and Bacteroides genera were the most common bacteriological microorganisms that had been isolated from FG patients in previous studies (15, 16). In this study, the bacteriological agents most frequently detected in the wound cultures corresponded to those reported in medical literature and included E. coli, as well as Streptococcus, Staphylococcus. In rare cases, Enterococcus and Pseudomonas species were isolated. In contrast, the results of wound culture were negative, with no causative agent detected, in 21 (28.8%) cases. This is probably due to an anaerobic microbial growth that could not be isolated using our culture conditions. 

No clear consensus exists on the most reliable clinical parameters for prediction of poor prognosis in FG patients (15). FGSI has been developed to facilitate the prediction of FG outcomes in affected patients. Laor et al. found that an FGSI score of >9 corresponded to 75% probability of a lethal outcome, while a score of ≤9 correlated with 78% survival probability (3). This threshold for the prediction of patient mortality was also confirmed in other studies (14, 17). In this study, the mean FGSI score in deceased cases was significantly higher, which was consistent with other studies (7, 8, 18). Notwithstanding the significantly higher mean FGSI score in the deceased patient group, only 47.6% of patients who died had FGSI>9. 

Yeniyol et al. found that deceased patients had elevated serum levels of urea, creatinine, alkaline phosphatase, and lactate dehydrogenase, and high leukocyte counts, while their levels of sodium, potassium, HCO3, haematocrit, total protein, and albumin were lower than the survivors'(14). Clayton et al. found that amongst various variables, a blood urea nitrogen level of higher than 50 mg/dl was the most significant factor in predicting FG mortality (19). In the study conducted by Tsung-Yen Lin, only serum creatinine, potassium and haematocrit levels showed notable association with the risk of patient mortality (9). In our study, respiratory rate, haematocrit, and HCO3 level were found to be significantly different between survivors and deceased patients.

Thrombocytopenia increased bleeding tendency and susceptibility to infection (20) and multiple studies pointed to the relationship between thrombocytopenia and likelihood of death in sepsis (21, 22). Mean platelet counts of survivors and deceased patients was in a normal range but deceased patients had significantly lower mean platelet counts (135400±77600/mm³ vs. 256700±136800/mm³, p = 0.0001). Platelet counts of seven patients were <50000/mm^3^, six of whom died. 

According to our findings, some of the nine parameters incorporated in FGSI were not significantly different between deceased and survived FG patients. However, some other factors including age, thrombocytopenia, and especially platelet counts <50000/mm^3^ were found to be predictive of mortality risk in these patients. Therefore, more studies using larger sample sizes are required to further evaluate these parameters in order to facilitate the development of a more efficacious FG scoring model.

## Limitation

One of the limitations of our study is that due to incomplete files and the retrospective fashion of the study, we had to exclude many cases before data analysis, so it reduced our sample size. Nevertheless, this sample size is one of the largest sample sizes for this rare disease.

## Conclusion:

The rate of mortality of FG and necrotizing fasciitis was 28.8% in this study. Based on multivariate analysis, the independent factors related to mortality were older age, lower haematocrit level and platelet count, and presence of immunosuppression.

## Author contribution

**AAD:** Conception and design, Critical revision of the manuscript for important intellectual content, Supervision 

**AR:** Analysis and Interpretation of data, Statistical analysis 

**BJ:** Administrative, technical or material support, Supervision 

**SM:** Drafting of the manuscript 

**VS:** Acquisition of data, Analysis and Interpretation of data 

**SAH:** Acquisition of data 

**SG: **Drafting of the manuscript 

**RH:** Drafting of the manuscript, Supervision 

**JK: **Conception and design, Critical revision of the manuscript for important intellectual content, Administrative, technical or material support Supervision 

All authors have read and approved the manuscript.

## Data availability

Authors guarantee that data of the study are available and will be provided if anyone needs them.

## Conflict of interests

None

## Funding

 None

## References

[1] Hsu J-M, Chen M, Weng C-H, Tseng J-S (2014). Fournier's gangrene: Clinical characteristics in the elderly. International Journal of Gerontology..

[2] Eke N (2000). Fournier's gangrene: a review of 1726 cases. British Journal of Surgery..

[3] Laor E, Palmer LS, Tolia BM, Reid RE, Winter HI (1995). Outcome prediction in patients with Fournier's gangrene. The Journal of urology..

[4] Spirnak JP, Resnick MI, Hampel N, Persky L (1984). Fournier’s gangrene: report of 20 patients. The Journal of urology..

[5] Medina Polo J, Tejido Sánchez A, De la Rosa Kehrmann F, Felip Santamaría N, Blanco Álvarez M, Leiva Galvis O (2008). Gangrena de Fournier: estudio de los factores pronósticos en 90 pacientes. Actas urológicas españolas..

[6] Jeong HJ, Park SC, Seo IY, Rim JS (2005). Prognostic factors in Fournier gangrene. International journal of urology..

[7] Luján Marco S, Budía A, Di Capua C, Broseta E, Cruz FJ (2010). Evaluation of a severity score to predict the prognosis of Fournier’s gangrene. BJU international..

[8] Corcoran A, Smaldone M, Gibbons E, Walsh T, Davies B (2008). Validation of the Fournier's gangrene severity index in a large contemporary series. The Journal of urology..

[9] Lin TY, Ou CH, Tzai TS, Tong YC, Chang CC, Cheng HL (2014). Validation and simplification of F ournier's gangrene severity index. International Journal of Urology..

[10] Tuncel A, Aydin O, Tekdogan U, Nalcacioglu V, Capar Y, Atan A (2006). Fournier’s gangrene: three years of experience with 20 patients and validity of the Fournier’s gangrene severity index score. European urology..

[11] Hazrati E, Noorifard M, Afsahi M, Rohi A, Rafiei M (2018). The relationship between lipid profile and CRP in severe sepsis with patient prognosis. Journal of Advanced Pharmacy Education & Research.

[12] Martinelli G, Alessandrino E, Bernasconi P, Caldera D, Colombo A, Malcovati L (1998). Fournier’s gangrene: a clinical presentation of necrotizing fasciitis after bone marrow transplantation. Bone marrow transplantation..

[13] Kuo C, Wang W, Lee C, Liu C, Tseng H (2007). Fournier's gangrene: ten-year experience in a medical center in northern Taiwan. Journal of Microbiology Immunology and Infection..

[14] Yeniyol CO, Suelozgen T, Arslan M, Ayder AR (2004). Fournier's gangrene: experience with 25 patients and use of Fournier's gangrene severity index score. Urology..

[15] Tuncel A, Keten T, Aslan Y, Kayali M, Erkan A, Koseoglu E (2014). Comparison of different scoring systems for outcome prediction in patients with Fournier's gangrene: experience with 50 patients. Scandinavian journal of urology..

[16] Saeidinia A, Keihanian F, Delavar SF, Keihanian F, Ranjbar A, Karkan MF (2016). Lack of antibacterial activity of Ruta graveolens extracts against Enterococcus fecalis. Pak J Pharm Sci..

[17] Chawla SN, Gallop C, Mydlo JH (2003). Fournier’s gangrene: an analysis of repeated surgical debridement. European urology..

[18] Kabay S, Yucel M, Yaylak F, Algin MC, Hacioglu A, Kabay B (2008). The clinical features of Fournier’s gangrene and the predictivity of the Fournier’s Gangrene Severity Index on the outcomes. International urology and nephrology..

[19] Clayton M, Fowler JJ, Sharifi R, Pearl R (1990). Causes, presentation and survival of fifty-seven patients with necrotizing fasciitis of the male genitalia. Surgery, gynecology & obstetrics..

[20] Yeaman MR (2019). The role of platelets in antimicrobial host defense.

[21] Venkata C, Kashyap R, Farmer JC, Afessa B (2013). Thrombocytopenia in adult patients with sepsis: incidence, risk factors, and its association with clinical outcome. Journal of intensive care..

[22] Stephan F, Montblanc J, Cheffi A, Bonnet F (1999). Thrombocytopenia in critically ill surgical patients: a case-control study evaluating attributable mortality and transfusion requirements. Critical Care..

